# Activity of Polyphenolic Compounds against *Candida glabrata*

**DOI:** 10.3390/molecules201017903

**Published:** 2015-09-29

**Authors:** Ricardo Salazar-Aranda, Graciela Granados-Guzmán, Jonathan Pérez-Meseguer, Gloria M. González, Noemí Waksman de Torres

**Affiliations:** 1 Departamento de Química Analítica, Facultad de Medicina, Universidad Autónoma de Nuevo León, Madero y Aguirre Pequeño, Col. Mitras Centro. Monterrey, N.L. C.P. 64460, Mexico; E-Mails: ricardo.salazarar@uanl.edu.mx (R.S.-A.); gracielagranadosg@yahoo.com.mx (G.G.-G.); jonathanmeseguer@hotmail.com (J.P.-M.); 2 Departamento de Microbiología, Facultad de Medicina, Universidad Autónoma de Nuevo León, Madero y Aguirre Pequeño, Col. Mitras Centro. Monterrey, N.L. C.P. 64460, Mexico; E-Mail: gloria62@hotmail.com

**Keywords:** *Candida glabrata*, polyphenols, flavonoids, myricetin, baicalein

## Abstract

Opportunistic mycoses increase the morbidity and mortality of immuno-compromised patients. Five *Candida* species have been shown to be responsible for 97% of worldwide cases of invasive candidiasis. Resistance of *C. glabrata* and *C. krusei* to azoles has been reported, and new, improved antifungal agents are needed. The current study was designed to evaluatethe activity of various polyphenolic compounds against *Candida* species. Antifungal activity was evaluated following the M27-A3 protocol of the Clinical and Laboratory Standards Institute, and antioxidant activity was determined using the DPPH assay. Myricetin and baicalein inhibited the growth of all species tested. This effect was strongest against *C. glabrata*, for which the minimum inhibitory concentration (MIC) value was lower than that of fluconazole. The MIC values against *C. glabrata* for myricitrin, luteolin, quercetin, 3-hydroxyflavone, and fisetin were similar to that of fluconazole. The antioxidant activity of all compounds was confirmed, and polyphenolic compounds with antioxidant activity had the greatest activity against *C. glabrata*. The structure and position of their hydroxyl groups appear to influence their activity against *C. glabrata*.

## 1. Introduction

The World Health Organization reported in 2004 that infectious diseases were among the top 10 causes of death in the general population and among the top five causes in children younger than 5 years [1]. However, few drugs are available, and most of them are associated with toxicity [2].

*Candida* species comprise one of the four main types of organisms that can be isolated from blood cultures of hospitalized patients and are the most important cause of opportunistic fungal infection worldwide [3]. The *Candida* genus comprises about 154 species, although 97% of invasive candidiasis is attributed mainly to five species: *C. albicans* (42.1%), *C. glabrata* (26.7%), *C. parapsilosis* (15.9%), *C. tropicalis* (8.7%), and *C. krusei* (3.4%) [3]. In Mexico, *C. glabrata* is the predominant species that causes vaginal mycosis [4]. The main challenge to the future development of prophylactic and therapeutic strategies is the resistance of species other than *C. albicans* to the available antifungal drugs. Resistance to azoles is a potential problem for treating infections caused by *C. glabrata*, *C. krusei*, and other less common species [5].

Considering the medical importance of the *Candida* species, we assessed the antifungal activity of a large number of extracts and compounds isolated from plants. We used clinical isolates of the major species that cause candidiasis as a study model. Recently, we reported on the antimicrobial activity of extracts from 17 plants that grow wild in the northeastern region of Mexico [6]. All plant extracts showed activity against at least one isolate of *Candida* species, of which *C. glabrata* was the most sensitive. In addition, >80% of extracts with activity against *C. glabrata* clearly showed high antioxidant activity. These plant extracts also had a high level of polyphenolic compounds, especially flavonoids.

Polyphenolic compounds are secondary metabolites that are widely distributed in the plant kingdom and are often consumed through beverages and foods made from the plants that contain them. Most polyphenols in plants are flavonoids, which contribute to the defense against plant pathogens and the response to changing environmental conditions [7]. The antimicrobial activity of flavonoids and their glycosylated derivatives has been investigated widely, and associations between their antimicrobial and antioxidant activities have been suggested [8]. However, little has been reported about the activity of certain flavonoids against strains of *C. glabrata*. We evaluated whether 18 polyphenolic compounds exerted inhibitory effects on the growth of *C. albicans*, *C. parapsilosis*, *C. tropicalis*, *C. krusei*, and *C. glabrata*.

## 2. Results and Discussion

Both baicalein and myricetin inhibited the growth of all species of yeast used in our study. Baicalein showed the greatest activity, although both were less active than the positive control, fluconazole (Table 1). The exception was *C. glabrata*, which was inhibited to a greater degree by these two compounds than by the control.

**Table 1:** MIC values of flavonoids on isolates of *Candida* species.

	*Candida* Isolate												
Flavonoid ^#^	*C. tropicalis* 166	*C. krusei* 168	*C. parapsilosis* 96	*C. albicans* 498	*C. albicans* 501	*C. albicans* 53	*C. albicans* ATCC 10231	*C. glabrata* 510	*C. glabrata* 493	*C. glabrata* 482	*C. glabrata* 531	*C. glabrata* 587	*C. glabrata* 507
Baicalein	10.4	21	5.2	10.4	10.4	10.4	10.4	1.9	1.9	1.9	1.9	1.9	1.9
Myricetin	64	64	16	64	32	16	ND	3.9	3.9	3.9	3.9	3.9	3.9
Myricitrin	>83	>83	>83	>83	>83	>83	>83	3.9	3.9	3.9	3.9	7.8	7.8
Luteolin	>83	>83	>83	>83	>83	>83	>83	7.8	3.9	3.9	7.8	7.8	7.8
Quercetin	>83	>83	>83	>83	>83	>83	>83	15.6	15.6	7.8	7.8	7.8	7.8
3-Hydroxyflavone	>83	>83	>83	>83	>83	>83	>83	3.9	7.8	15.6	15.6	15.6	3.9
Fisetin	>83	>83	>83	>83	>83	>83	>83	7.8	7.8	7.8	7.8	15.6	15.6
Quercitrin	>83	>83	>83	>83	>83	>83	>83	62.5	31.2	7.8	7.8	7.8	7.8
Kaempferol	>83	>83	>83	>83	>83	>83	>83	31.2	31.2	31.2	31.2	31.2	31.2
Galangin	>83	>83	>83	>83	>83	>83	>83	31.2	31.2	15.6	31.2	31.2	62.5
Flavone	>83	>83	>83	>83	>83	>83	>83	62.5	62.5	62.5	62.5	62.5	62.5
Fluconazole	1.9	3.9	0.9	0.9	0.5	3.9	0.5	1.9	3.9	3.9	31.2	7.8	3.9

^#^ MIC values for each flavonoid are listed as µg/mL; ND, not determined. 5-hydroxyflavone, 6-hydroxyflavone, 7-hydroxyflavone, chrysin, apigenin, naringenin, and hesperetin showed no effect on the growth of *Candida* species at the highest concentration used (83 µg/mL).

Eleven compounds showed growth-inhibiting activity against the six clinical isolates of *C. glabrata*. Baicalein and myricetin showed activity against all clinical isolates of *C. glabrata*, and this activity was equivalent to or greater than that of fluconazole (Table 1). Myricitrin, luteolin, quercetin, 3-hydroxyflavone, and fisetin caused strong inhibition of the growth of *C. glabrata* and had minimum inhibitory concentration (MIC) values similar to those of the positive control. Quercitrin, kaempferol, galangin, and flavone also showed inhibitory activity against *C. glabrata* growth, although their MIC values were higher than those of the positive control. Clinical isolate 531, which was resistant to fluconazole, was susceptible to at least seven of the flavonoids studied.

Eight of the 18 compounds tested showed good antioxidant activity; their median effective concentration values (EC_50_) were <20 µM (Table 2).

**Table 2:** EC_50_ for reduction of 2,2-diphenyl,1-picrylhydrazyl (DPPH).

Flavonoid	MW	EC_50_ (µM)	Previously Reported (µM)	Reference
Luteolin	286.2	3.46	22.8	[9]
			1.8	[10]
Fisetin	286.2	5.34	11.8	[11]
			1.1	[10]
Myricetin	318.2	9.1	12.3	[11]
			1.6	[10]
Quercetin	302.2	10.6	2.2	[11]
			8.1	[10]
			9.7	[12]
Kaempferol	286.2	11.6	25.7	[11]
			7.2	[10]
Baicalein	270.2	17.6	5.1	[10]
Myricitrin	464.4	11.6	12.7	[13]
Quercitrin	448.4	12.5	11	[12]
Galangin	270.2	426	185.05	[14]
			11	[10]
3-Hydroxyflavone	238.2	525	>200	[10]
Hesperetin	302.3	>413		
Naringenin	272.2	>459	>200	[10]
5-Hydroxyflavone	238.2	>525		
Flavone	222.1	>563	>200	[10]
Chrysin	254.2	>492	>200	[10]
7-Hydroxyflavone	238.2	>525		
6-Hydroxyflavone	238.2	>525		
Apigenin	270.2	>463	>200	[10]

EC_50_, 50% effective concentration; MW, molecular weight.

Because of the capacity of flavonoids to inhibit spore germination of pathogens in plants, flavonoids have been proposed for use against fungal pathogens in humans [15]. Several authors have reported on the ability of flavonoids and flavonoid derivatives to inhibit the growth of *C. albicans* [16,17]. In this work, we found strong activity of two flavonoids against *Candida* species: baicalein, whose MIC values were 5.2–21 µg/mL, and myricetin, whose MIC values were 16–64 µg/mL. These values obtained using baicalein agree with those recently reported by Serpa [16] against *C. albicans*, *C. tropicalis*, and *C. parapsilosis*. Li *et al.* [18] evaluated the effects of myricetin on the growth of fungi, yeast, and bacteria, among them *C. albicans*, although they did not report the effects of this compound on the growth of *C. albicans*. Li *et al.* used Sabouraud’s dextrose broth, whereas we used RPMI-1640 medium; several authors have reported that the medium composition can affect susceptibility to antifungal agents [19].

The most interesting result in our study was that a large number of flavonoids exerted inhibitory activity against clinical isolates of *C. glabrata*, which has not been reported previously for any of these flavonoids. These results are particularly important considering that vaginal candidiasis in Mexico is caused mainly by *C. glabrata*. This yeast is persistent and is resistant to fluconazole, the most widely used antifungal drug [4]. There is limited information in the literature about the effects of polyphenolic compounds on the inhibition of growth of *C. glabrata* [20,21]. We report here that eight flavonoids had strong activity; two are flavones and the other six are free or glycosylated flavonols. The presence or absence of a sugar moiety did not seem to influence the activity against *C. glabrata* (e.g., compare the activity of myricitrin *vs.* myricetin, and quercetin *vs.* quercitrin).

All of the flavonols and two flavones that we analyzed were active against *C. glabrata*, but the activity was markedly greater for compounds that showed two *ortho*–OH groups. The hydroxylation pattern on the B or C ring of the flavonoid may determine the degree of activity, and catechol structures may be the most active (e.g., compare apigenin *vs.* luteolin and quercetin *vs.* kaempferol). The exception was 3-hydroxyflavone.

Considering that this structural requirement is similar to what has been reported for the antioxidant activity of flavonoids [22,23], in the next step, we wanted to study further pure compounds with an EC_50_ > 125 µg/mL, which could be regarded as indicating inactivity. Using our current results, we divided the flavonoids into those with high free radical-scavenging activity (EC_50_ < 20 µg/mL) and those with little or no antioxidant capacity (EC_50_ > 100 µg/mL). Interestingly, the compounds with greater antioxidant activity (EC_50_ < 20 µg/mL) also showed activity against *C. glabrata*, with only kaempferol as the exception.

Silva described three important requirements of a flavonoid for being a good free radical scavenger: (1) an *o*-catechol B-ring group, which is regarded as the key feature; (2) a double bond in C_2_–C_3_, which ensures B-ring conjugation with the 4-oxo group; and (3) –OH groups at C_3_ and C_5_, which allow the delocalization of the 4-oxo group to both substituents [24]. The combination of these factors allows a greater electron delocalization. Although kaempferol lacks the first characteristic, it has the second and third characteristics and may function as a free radical scavenger. Most previous studies have focused on the catechol structure in the B ring, and little research has been done on the A ring. Marković recently performed structure-activity studies and demonstrated the importance of the A ring, which determines the antioxidant activity of baicalein [25]. Our results are consistent with these other findings.

The presence of a third –OH group at the *ortho* position to the catechol structure exerts little influence on the antioxidant capacity of the flavonoid, as shown by the results obtained with quercetin and myricetin. However, this third –OH group seemed to influence the activity against *C. glabrata*, because the two compounds with greater activity (*i.e.*, myricetin and baicalein) have a pattern of hydroxylation with three –OH groups on neighboring carbons (Table 3), although one is a flavone and the other a flavonol.

Baicalein, which is obtained from *Scutellaria baicalensis*, has been shown in pharmacological studies to have antioxidant, neuroprotective, antimicrobial, and antiviral activities [26,27]. Baicalein also has antifungal activity, such as the ability to inhibit the growth of *C. albicans*, and exhibits synergism with fluconazole [28,29]. A recent report showed that baicalein can induce apoptosis of *C. albicans* [30]. Another report has shown that baicalein has activity against a clinical isolate of *C. krusei*, which is fluconazole resistant, at a level that is superior to that observed against *C. albicans* [31]. Our report is the first to show an inhibitory effect of baicalein on clinical isolates of fluconazole-resistant *C. glabrata* and that its effectiveness was five times higher than that observed against *C. albicans*. Myricetin, the other flavonoid showing high activity against *C. glabrata*, was also 4 to 16 times more effective than against *C. albicans*. To our knowledge, no reports of the activity of this flavonoid against *Candida* species have been published.

Interestingly, most flavonoids with activity against *C. glabrata* are found at high concentration in natural sources with broad use, such as *S. baicalensis* [32], *Oroxylum indicum* [33], berries [34], capers [35], and other sources*.* Further studies of the mechanisms of action of these flavonoids and products containing these compounds may indicate whether it might be possible to generate low-cost drugs against candidiasis caused by this organism.

**Table 3:** Polyphenols structure. 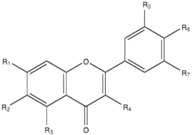

Polyphenols	R_1_	R_2_	R_3_	R_4_	R_5_	R_6_	R_7_
Flavone	–H	–H	–H	–H	–H	–H	–H
3-Hydroxyflavone	–H	–H	–H	–OH	–H	–H	–H
5-Hydroxyflavone	–H	–H	–OH	–H	–H	–H	–H
6-Hydroxyflavone	–H	–OH	–H	–H	–H	–H	–H
7-Hydroxyflavone	–OH	–H	–H	–H	–H	–H	–H
Chrysin	–OH	–H	–OH	–H	–H	–H	–H
Apigenin	–OH	–H	–OH	–H	–H	–OH	–H
Baicalein	–OH	–OH	–OH	–H	–H	–H	–H
Luteolin	–OH	–H	–OH	–H	–OH	–OH	–H
Galangin	–OH	–H	–OH	–OH	–H	–H	–H
Kaempferol	–OH	–H	–OH	–OH	–H	–OH	–H
Fisetin	–OH	–H	–H	–OH	–OH	–OH	–H
Quercetin	–OH	–H	–OH	–OH	–OH	–OH	–H
Myricetin	–OH	–H	–OH	–OH	–OH	–OH	–OH
Quercitrin	–OH	–H	–OH	–ORha	–OH	–OH	–H
Myricitrin	–OH	–H	–OH	–ORha	–OH	–OH	–OH

## 3. Experimental Section

### 3.1. Chemicals

Flavone, 5-hydroxyflavone, 6-hydroxyflavone, 7-hydroxyflavone, chrysin, apigenin, baicalein, luteolin, naringenin, hesperetin, 3-hydroxyflavone, galangin, kaempferol, quercetin, fisetin, myricetin, RPMI-1640, dimethylsulfoxide (DMSO), methanol, and 2,2-diphenyl,1-picrylhydrazyl (DPPH) were purchased from Sigma-Aldrich (St Louis, MO, USA). Fluconazole (Diflucan) was obtained as an intravenous solution at 2 mg/mL from Pfizer S.A. de C. V (Toluca de Lerdo, Mexico). Myricitrin and quercitrin were isolated and purified from *Juglans mollis* cortex, and their purity was verified by thin-layer chromatography and High Performance Liquid Chromatography. The structures were confirmed by the ^1^H-NMR and ^13^C-NMR spectra [36]. The flavonoid structures are shown in Table 3 and Table 4.

**Table 4:** Flavanones structure. 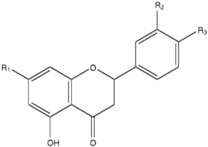

Flavanones	R_1_	R_2_	R_3_
Naringenin	–OH	–H	–OH
Hesperetin	–OH	–OH	–OCH_3_

### 3.2. Antifungal Activity

Antifungal activity was evaluated using 12 clinical isolates as study models of sensitive strains of yeast (the isolate numbers are given in parentheses): *C. parapsilosis* (96), *C. tropicalis* (166), *C. krusei* (168), *C. albicans* (501, 498, 53, and ATCC 10231), and *C. glabrata* (507, 531, 587, 510, 493, 482). All clinical isolates were obtained from cultures of blood, cerebrospinal fluid, and nails, and each *Candida* species was identified through the use of API 20C AUX strips (bioMérieux, México, D.F., Mexico) and standard morphological methods, in the Department of Microbiology, Faculty of Medicine, Autonomous University of Nuevo León. Each compound was evaluated at concentrations between 0.15 and 83 µg/mL. The MIC values were determined by microdilution in 96-well plates using RPMI-1640 and visual detection following protocol M27-A3 of the Clinical and Laboratory Standards Institute [37]. All assays were performed in duplicate, and sterility controls and growth controls without and with fluconazole (Diflucan) as blank and positive controls were included.

### 3.3. Free Radical Reduction: DPPH Assay

Antioxidant activity was determined according to the method reported by Garza [38]. Solutions of each compound in ethanol were serially diluted with additional ethanol to concentrations in the range of 125 to 0.5 µg/mL. The DPPH solution was prepared at a concentration of 125 µM. Each diluted compound or blank (500 µL) was placed individually in a test tube, and 0.5 mL of DPPH was added. The solutions were stirred and allowed to react in the dark for 30 min. Spectrophotometric measurement was performed at a wavelength of 517 nm. The reduction of DPPH was calculated from the following equation:

% Reduction = [(Blank − Sample)/Blank] × 100(1)

We created a linear regression curve with the percent reduction as a function of the concentration of each compound and calculated the concentration that effectively reduced DPPH by 50% (EC_50_). All experiments were performed in triplicate, and the mean and standard deviation were calculated in each case.

## 4. Conclusions

We found significant antifungal activity of a number of flavonoids, especially baicalein and myricetin, against clinical isolates of fluconazole-resistant *C. glabrata*. Flavonoids displaying greater antioxidant activity were the most active against *C. glabrata*. *C. albicans* was less susceptible to these flavonoids.
